# Functional Links Between Leaf Traits, Soil Properties, and Aboveground Biomass Along an Altitudinal Gradient in Chitwan, Nepal

**DOI:** 10.1002/ece3.74171

**Published:** 2026-08-12

**Authors:** Shakti Raj Giri, Purnima Regmi, Rashila Deshar, Ramesh Prasad Sapkota

**Affiliations:** ^1^ Central Department of Environmental Science Institute of Science and Technology, Tribhuvan University Kirtipur Nepal

**Keywords:** biomass, diversity, environmental factors, leaf traits, plant species

## Abstract

Altitudinal gradients strongly influence plant species diversity and functional trait expression in forest ecosystems. This study aimed to investigate how leaf traits and environmental variables affect aboveground tree biomass along an altitudinal gradient in Chitwan District, Nepal. We established 90 circular plots (radius 11.3 m), with 30 plots in each of the three altitudinal bands: low (150–600 m), mid (600–1500 m), and high (1500–1930 m). Leaf traits, including leaf thickness, leaf area, specific leaf area (SLA), leaf dry matter content (LDMC), total chlorophyll content, total nitrogen, and phosphorus were measured for nine dominant tree species: *Shorea robusta* , *Trewia nudiflora*, *Cleistocalyx operculatus*, *Terminalia alata*, *Diploknema butyracea* , *Schima wallichii*, *Rhododendron arboreum*, *Quercus semecarpifolia*, and *Quercus lanata*. Mid‐elevation exhibited the highest tree species richness and diversity. Resource‐acquisitive traits (SLA, leaf area, nutrient content, and chlorophyll) were positively associated with biomass, whereas structural traits (LDMC and leaf thickness) were negatively correlated. Structural equation modeling (SEM) revealed that integrated leaf traits had a significant positive effect on biomass accumulation (β = 0.504, *p* < 0.001). Elevation and soil properties together explained 45.4% of the variation in leaf functional traits and 30.8% of the variation in the above‐ground biomass. These trait–environment relationships reveal adaptive strategies across altitudinal gradients, and provide insights for sustainable forest management under changing environmental conditions.

## Graphical abstract

Resource‐acquisitive leaf traits enhance aboveground biomass accumulation, whereas structural traits constrain it. Elevation and soil properties shape trait expression and biomass across the gradients.

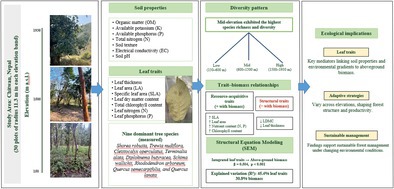

## 1 Introduction

Traits are quantifiable morphological, physiological, or phenological features of an individual (Violle et al. 2007). Functional traits (FTs) are a subset of traits that are more or less closely related and have a direct or indirect effect on plant fitness through their effect on growth, reproduction, or survival and, as a consequence, on community structure and ecological processes (Violle et al. 2007; Garnier et al. 2016). The traits have increasingly emerged as crucial proxies with which to make sense of species strategies, vegetation dynamics, and ecosystem function independently of simple taxonomic classification (Violle et al. 2007; Díaz et al. 2016). Functional traits underpin how plants access, use, and retain resources and impact productivity, nutrient cycling, and resilience to environmental change (Reich 2014; Garnier et al. 2016).

Functional trait‐based methods are becoming increasingly central in ecological and evolutionary studies, offering a robust framework for evaluating how plant species influence ecosystem activities and react to environmental gradients (Violle et al. 2007; Díaz et al. 2016). These traits are essential for predicting community assembly because they are influenced by selective pressures from environmental filters (e.g., temperature, soil nutrients, and water availability) and by disturbance frequency, intensity, and extent. Environmental filtering processes favor specific trait combinations that allow species to persist under local conditions while excluding others (Keddy 1992; Laughlin 2014). These interactions between traits and the environment form the basis of trait‐based ecology. Traits can serve a dual purpose, viz., response traits and effect traits (Lavorel and Garnier 2002; Nock et al. 2016). The traits that determine a species impact on ecosystem qualities and, consequently, the benefits or drawbacks that human civilizations experience are known as effect traits. Alternatively, response traits influence a species ability to adapt to environmental changes and colonize or thrive in a habitat (Díaz et al. 2013; Nock et al. 2016).

The plant functional traits are generally classified according to the organs of the plant: whole plant traits, stem traits, belowground traits, regenerative traits, and leaf traits are all important indicators of plant ecological strategies (Cornelissen et al. 2003; Pérez‐Harguindeguy et al. 2013). Leaf traits are one of these important plant characteristics since they play a key role in photosynthesis, nutrient turnover, water regulation, and energy fluxes of plants to and from the abiotic environment (Violle et al. 2007; Pérez‐Harguindeguy et al. 2013). Different traits, including SLA, LDMC, chlorophyll concentration, and leaf nitrogen and phosphorus contents, are important to understanding the ecological strategies of species and their adaptive responses to environmental change (Reich et al. 1999; Wright et al. 2004). To show trade‐offs between conservation and rapid resource acquisition, the leaf economics spectrum (LES) synthesizes these functional relationships (Wright et al. 2004). Low SLA species prioritize longevity, stress tolerance, and tissue toughness, whereas high SLA species with nutrient‐rich leaves typically exhibit high photosynthetic capacity, rapid growth, and short lifespans (Wright et al. 2004; Díaz et al. 2016). Leaf features are also closely linked to ecological activities such as carbon uptake, biomass accumulation, and nutrient cycling (Poorter et al. 2009; Garnier et al. 2016).

Species composition, climate, soil characteristics, and plant functional traits are some of the variables that affect biomass accumulation, which is defined as the net increase in organic matter during a certain time period (Poorter et al. 2015). It is a key measure of carbon storage and ecosystem productivity and is heavily impacted by the functional traits of plants (Reich 2014). Traits such as SLA, leaf nutrient content, and wood density play a significant role in regulating photosynthesis, nutrient uptake, biomass allocation, and growth efficiency (Reich 2014; Augusto and Boča 2022). According to Wright et al. (2004), species with conservative traits like high LDMC and wood density prioritize persistence and resource conservation above quick growth, whereas species with acquisitive traits like high SLA and leaf nitrogen generally show rapid biomass gain under ideal conditions.

Altitudinal gradients provide a natural framework for understanding how climate change impacts plant characteristics and ecosystem activity. As elevation increases, temperature decreases progressively, accompanied by changes in solar radiation intensity, precipitation patterns, wind exposure, and soil nutrient availability (Körner 2007; Niu et al. 2019). These abiotic shifts create strong selective pressures that drive changes in plant morphology, physiology, phenology, and community composition. These trait shifts also significantly affect primary productivity, biomass allocation, and carbon cycling across elevation bands (Körner 2007; Dieleman et al. 2013; Niu et al. 2019).

Along environmental gradients, trait complexity impacts ecosystem processes and community composition by reflecting ecological strategies from resource acquisition to conservation (Wright et al. 2004; Bruelheide et al. 2018). Along elevation gradients, functional traits such as nutrient concentrations, leaf thickness, and SLA often exhibit systematic fluctuations. To minimize water loss and enhance resource conservation in cold, nutrient‐limited environments, plants at high elevations typically develop denser, thicker leaves with a lower SLA (Read et al. 2014; Midolo et al. 2019). Reduced phosphate and nitrogen content in leaves is also common, indicating lower metabolic rates and decreased food availability in colder regions (Tang et al. 2018; Guo et al. 2020). These changes in traits are closely linked to shifts in carbon allocation and productivity, which influence biomass accumulation at the ecosystem level (Kitayama and Aiba 2002; Santiago and Wright 2007).

The study area is located within the Chitwan–Annapurna Landscape, one of the Nepal's ecologically important regions, encompassing tropical and subtropical forest ecosystems. Vegetation changes predictably along the elevational gradient, from lowland *Shorea robusta* (Sal) forests to mixed broadleaf forests dominated by *Schima wallichii*, *Castanopsis indica*, and associated tree species (Stainton 1972; Pokhrel and Sherpa 2020). This vegetation transition reflects changes in environmental conditions and contributes to the high floristic diversity characteristic of the central Himalayan region (Bhattarai and Vetaas 2006; Maharjan et al. 2021).

Plant communities along altitudinal gradients exhibit systematic variation in functional traits due to changes in temperature, moisture, and soil nutrient availability (Körner 2007; Sundqvist et al. 2013). A functional trait‐based approach provides a useful framework for understanding how plants respond to these environmental gradients and how such responses influence ecosystem processes such as biomass accumulation (Lavorel and Garnier 2002; Díaz et al. 2016). In this study, we hypothesize that (i) plant functional traits will shift along the altitudinal gradient from acquisitive to conservative strategies, and (ii) soil properties, particularly nutrient‐related variables, will significantly influence these trait variations and, consequently, aboveground biomass.

While trait–biomass relationships are well established within frameworks such as the leaf economics spectrum, integrative studies combining leaf traits, soil properties, and biomass along altitudinal gradients remain limited, particularly in the forests of Nepal. This study addresses this gap by examining how edaphic conditions and plant functional traits jointly influence biomass accumulation, with a focus on system‐specific patterns across the altitudinal gradient.

## 2 Materials and Methods

### 2.1 Study Area

**FIGURE 1:**
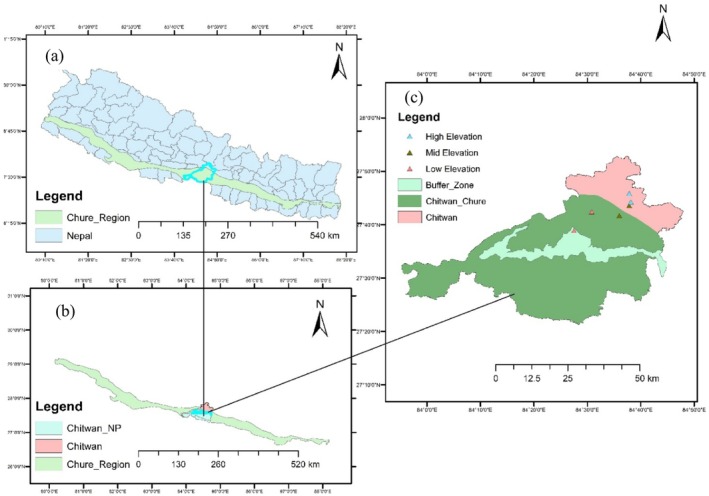
Map of study area showing (a) Nepal and Chure Region, (b) CNP, Chitwan, and Chure Region, and (c) sampling elevation bands with respective sites.

The study was conducted in the Chure Region and adjoining Siraichuli Hill of Chitwan District, central Nepal. The lower elevation sites (150–600 m a.s.l.) are located within Chitwan National Park, a globally recognized protected area that supports extensive *Shorea robusta* ‐dominated forests and high biodiversity. The Chure (Siwalik Hills), the southernmost range of the Himalayas, is a geologically young and ecologically sensitive landscape characterized by fragile sedimentary formations, steep slopes, erosion risks, and shallow soils (Uprety et al. 2023). The study area extends from the lowland Chure foothills (~150 m a.s.l.) to the Siraichuli Hill region of the Mahabharat Range (~1930 m a.s.l.) (Figure 1), encompassing substantial variation in climate, soil conditions, and vegetation composition.

The region experiences pronounced variation in temperature and precipitation along the elevation gradient. Lower Chure sites within Chitwan National Park experience warm, humid conditions, with mean annual temperatures of approximately 20°C–25°C and annual precipitation of around 1500 mm, which occurs primarily during the summer monsoon (June–September). In contrast, higher elevations of the Siraichuli Hill region (1500–1930 m a.s.l.) are cooler and more humid, with mean annual temperatures decreasing to approximately 12°C–15°C due to elevation effects and orographic influences (MoFE 2018).

Soil conditions vary along the elevational gradient due to differences in parent material, topography, and vegetation cover (Körner 2007; Sundqvist et al. 2013). The lower elevation sites are characterized by predominantly alluvial and colluvial soils with relatively higher moisture and nutrient availability. In contrast, soils of the Chure (Siwalik) region are derived from young sedimentary rocks and are generally coarse‐textured, shallow, highly permeable, nutrient‐poor, and have low water‐holding capacity (Uprety et al. 2023; Dijkshoorn and Huting 2009). At higher elevations toward the Siraichuli Hill region, cooler temperatures and denser forest cover promote greater soil organic matter accumulation and slower rates of organic matter decomposition, resulting in relatively deeper and more developed soils (Sundqvist et al. 2013; Dijkshoorn and Huting 2009).

The vegetation of the study area varies along the elevation gradient. The lower elevation sites are dominated by Himalayan subtropical broadleaf forests, with *Shorea robusta* forming the major canopy species. In the Chure region, Sal forests occur with associated species such as *Pinus roxburghii* and other broadleaf trees, whereas higher elevations toward the Siraichuli Hill region support mixed broadleaf forests. These vegetation differences correspond with changes in climatic and edaphic conditions along the elevation gradient.

The study area experiences varying levels of anthropogenic disturbance, including fuelwood extraction, grazing, and localized land‐use pressure, which may influence vegetation structure and plant functional traits. Altitudinal zones used in this study were defined by elevation ranges, associated environmental variation, and observable changes in vegetation composition, ensuring ecological and methodological consistency.

### 2.2 Sampling Design and Sample Collection

#### 2.2.1 Vegetation Survey and Soil Sampling

The study employs a systematic sampling approach across an altitudinal gradient spanning 150–1930 m above sea level in the Chitwan District, Nepal. The study site was divided into three altitudinal bands, viz. low elevation (150–600 m), mid‐elevation (600–1500 m), and high elevation (1500–1930 m). The study utilized tree‐level data collected from 90 circular nested plots (30 plots in each band). Circular plots with a radius of 11.3 m were used for field sampling of trees, and a radius of 1 m was used for soil sampling. These plot sizes are based on the guidelines provided by FRTC (2022), with slight modifications made to the radii to better align with the specific conditions and objectives of this study. Data on all trees and saplings with a diameter at breast height (DBH) larger than 5 cm were collected. The DBH at 1.37 m above ground level and the height of each plant were measured using a DBH tape and a rangefinder (Nikon Forestry Pro II Laser Rangefinder), respectively. The number of seedlings within the plots was counted. Additionally, each plot's geographical coordinates, altitude, and canopy coverage were recorded using a GPS (Garmin GPSMAP 64s) and a densiometer (Spherical Densiometer Model A), respectively. Although sampling was conducted across a broad elevational gradient to capture environmental variation, potential spatial autocorrelation among plots was not explicitly assessed.

Plant species were first identified using the handbook of flowering plants of Nepal (Shrestha et al. 2018) and their endemic status was subsequently verified by cross‐referencing with published accounts of Nepalese endemic species provided by Rajbhandari et al. (2021).

Soil samples were collected from 0 to 10 cm, 10 to 20 cm, and 20 to 30 cm depths using a soil core sampler following the field sampling protocol described by the Forest Research and Training Centre (FRTC 2022). The upper 30 cm soil layer was selected because it represents an important zone of fine‐root activity and nutrient availability influencing plant functional traits and biomass production (Jackson et al. 1996; Jobbágy and Jackson 2001). Soil samples from different depths within each plot were mixed thoroughly to obtain a composite sample representing site‐level soil conditions. The collected samples were air‐dried after removing stones, roots, plant debris, and other unwanted particles before laboratory analysis.

#### 2.2.2 Leaf Traits Sampling

##### 2.2.2.1 Selection of Plants

In each plot, the most abundant species were selected to represent the plant community. Species selection followed standardized trait‐based sampling guidelines (Cornelissen et al. 2003; Pérez‐Harguindeguy et al. 2013), which recommend focusing on dominant species that together account for approximately 70%–80% of community biomass or abundance. This approach ensures a representative characterization of ecosystem functioning and allows upscaling of functional traits from species to community level. This is also consistent with the Mass Ratio Hypothesis (Grime 1998), which proposes that ecosystem processes are primarily driven by the traits of the most abundant species. In cases where biomass data were not directly available, species dominance was estimated based on the relative abundance of individuals within each plot. In the present study, the selected dominant species collectively represented approximately 70%–85% of total individual abundance within each altitudinal zone, ensuring that the most ecologically significant species were included in the analysis.

##### 2.2.2.2 Trait Selection

For trait measurements, healthy, fully expanded leaves were collected from well‐grown individuals, preferably under unshaded conditions, to minimize microenvironmental variation. For each selected species, 2–5 individuals per plot were sampled, and from each individual, 20–25 fully expanded, mature leaves were collected for trait measurements. Individuals showing signs of herbivory, disease, or physical damage were excluded following standardized plant functional trait protocols (Cornelissen et al. 2003; Pérez‐Harguindeguy et al. 2013). Sampling individuals were selected across the elevation range within each altitudinal zone, and where applicable, variation in slope aspect (north‐ and south‐facing slopes) was considered to reduce microclimatic bias.

Traits related to aboveground biomass were selected based on their ecological relevance and applicability across all species and sampling plots, representing key functional dimensions such as resource acquisition, structural support, and physiological performance. The selected traits, along with their descriptions and units, are presented in Table 1.

**TABLE 1:** Description of leaf traits measured in the study (LI = light interception, MS = mechanical support, and PD = physical defense).

Leaf traits	Abbreviation	Unit	Functions
Leaf thickness	LT	mm	MS
Leaf area	LA	cm^2^	LI
Specific leaf area	SLA	cm^2^/g	LI
Leaf dry matter content	LDMC	g/g	PD
Leaf chlorophyll content	TCH	mg/g	LI
Leaf N concentration	TN	%	MS
Leaf P concentration	TP	%	MS

### 2.3 Data Analysis

#### 2.3.1 Diversity Indices

Species diversity was quantified using the Hill numbers method, which provides a unified and mathematically consistent approach for measuring diversity across different diversity orders (Chao et al. 2014, 2016). Diversity orders q0, q1, and q2 were calculated, representing species richness, the effective number of common species (exponential Shannon diversity), and the effective number of dominant species (inverse Simpson diversity), respectively. Sample coverage was estimated to evaluate sampling completeness among elevation zones.

#### 2.3.2 Trait Measurement and Calculation

The protocols for measuring leaf traits recommended by Cornelissen et al. (2003) were followed. Table 2 shows the data analysis method of selected traits.

**TABLE 2:** List of leaf traits, measurement procedures and corresponding references.

Trait	Method summary	References
LA	Scanned fresh leaves were measured using ImageJ software	Schneider et al. 2012
SLA	Ratio of fresh LA to oven‐dry mass; leaves were dried at 60°C for 72 h	Cornelissen et al. 2003
Thickness	Measured at mid‐leaf using a digital caliper and averaged per species	Cornelissen et al. 2003
LDMC	Ratio of oven dry mass to fresh mass	Cornelissen et al. 2003
Chlorophyll	Acetone extraction, centrifuged, and absorbance was measured at 645 nm and 663 nm using a spectrophotometer	Arnon 1949
Leaf P	Acid digestion with absorbance measured at 410 nm	Estefan et al. 2013
Leaf N	Kjeldahl method, which includes digestion, distillation, and titration with HCl	Kjeldahl method (Bremner 1960)

#### 2.3.3 Biomass Estimation

To calculate the aboveground tree biomass, the ecological conditions of the forest were considered, and a biomass equation developed for moist forest stands was adopted, as suggested by Chave et al. (2005). Species‐specific wood density (*ρ*) values were used when available; otherwise, genus‐level or community‐average values were applied. The general allometric model used was

$$\text{AGTB}=0.0509\times D^{2}\times H\times \rho$$

where AGTB is the aboveground tree biomass (kg), ρ is the wood‐specific gravity (g/cm^3^), D is the tree diameter at breast height (cm), and H is the tree height (m).

##### 2.3.3.1 Soil Parameters

The analysis of soil parameters was carried out using standard procedures. Organic matter content was determined by the Walkley and Black method (Gupta 2002; Broadbent 2015), while available potassium concentration was measured using a flame photometer (Thomas 1982). Available phosphorus was analyzed through the Modified Olsen's bicarbonate method (Gupta 2002), and total nitrogen was derived using the Kjeldahl method (Bremner 1960). Soil texture at each elevation was assessed by the hydrometer method (Bouyoucos 1962).

### 2.4 Statistical Analysis

Statistical analyses were performed using R version 4.3.3 (R Core Team 2024). Prior to analysis, data were assessed for normality using the Shapiro–Wilk test and visual inspection of Q–Q plots, and homogeneity of variance was examined where appropriate. As several leaf traits and biomass variables deviated from normality assumptions, nonparametric methods were used for subsequent analyses. Differences in leaf traits and aboveground biomass among species and elevation bands were evaluated using the Kruskal–Wallis test, followed by Dunn's post hoc test with Benjamini–Hochberg adjustment for pairwise comparisons using the FSA package (Ogle et al. 2026).

To assess overall multivariate differences in functional trait composition among elevation bands, Permutational Multivariate Analysis of Variance (PERMANOVA) based on Euclidean distance matrices was performed using the adonis2 function in the vegan package (Oksanen et al. 2016). Homogeneity of multivariate dispersion was evaluated using the betadisper function to ensure that PERMANOVA results were not solely influenced by differences in within‐group variability. When the overall PERMANOVA was significant, pairwise PERMANOVA comparisons were conducted to identify differences among elevation bands. Principal component analysis (PCA) was subsequently conducted using the FactoMineR and factoextra packages (Lê et al. 2008; Kassambara and Mundt 2020) to summarize functional trait variation across elevation bands. Interpretation was based on trait loadings and the proportion of variance explained by the principal components. Relationships between leaf traits, and aboveground biomass were examined using Spearman's rank correlation. Simple linear regression analyses were used to evaluate the strength and direction of significant trait–biomass relationships.

To investigate the influence of environmental factors on trait variation and biomass production, Redundancy analysis (RDA) was performed using the vegan package, with soil properties as explanatory variables and leaf traits and biomass as response variables. Elevation was not included simultaneously with soil variables to avoid redundancy because it represents an integrated environmental gradient influencing climate, vegetation composition, and soil characteristics.

Hierarchical cluster analysis was used to classify species based on similarities in functional traits. Spearman rank correlation analysis was performed to evaluate relationships among environmental variables, leaf traits, and above‐ground biomass. Structural Equation Modeling (SEM) was conducted using the lavaan package (Rosseel 2012) to evaluate direct and indirect pathways linking functional traits and above‐ground biomass.

All statistical tests were evaluated at a significance level of *α* = 0.05, and results with *p* < 0.05 were considered statistically significant.

## 3 Results

### 3.1 Vegetation Analysis

The composition and diversity of vegetation varied along the elevation gradient. A total of 12, 16, and 10 tree species were recorded in the low, mid, and high elevation bands, respectively (Table 3). Species diversity patterns varied among elevation bands. Mid‐elevation sites showed the highest effective species diversity, with the greatest species richness (q0 = 16) and effective number of common species (q1 = 7.82), indicating higher community diversity and heterogeneity. High‐elevation sites exhibited relatively higher effective diversity of dominant species (q2 = 5.53), suggesting a more even distribution of abundant species. In contrast, low‐elevation sites showed lower diversity values (q1 = 2.29; q2 = 1.51), reflecting greater dominance by fewer species (Table 3).

**TABLE 3:** Species diversity patterns based on Hill numbers (q0, q1, and q2) across elevation bands.

Elevation band	Species richness (q0)	Effective number of common species (q1)	Effective number of dominant species (q2)
Low elevation	12	2.29	1.51
Mid elevation	16	7.82	5.29
High elevation	10	7.00	5.53

*Shorea robusta* , *Cleistocalyx operculatus*, *Trewia nudiflora*, *Cassia fistula* , *Aegle marmelos*, and *Garuga pinnata* were found in the low elevation band. In the mid‐elevation band, *Diploknema butyracea* , *Schima wallichii*, *Toona ciliata* , *Terminalia alata*, *Albizia julibrissin* , *Bauhinia purpurea* , *Mallotus philippensis* , and *Bombax ceiba* were recorded. Similarly, *Quercus lanata*, *Quercus semecarpifolia*, *Rhododendron arboreum*, *Myrica esculenta*, *Lyonia ovalifolia*, and *Castanopsis tribuloides* were recorded in the high elevation band.

### 3.2 Selected Species for Traits Analysis

For the study of plant functional traits, the most abundant tree species recorded in each plot were selected as representative species for further analysis. For traits measurement, the species selected were *Shorea robusta* , *Cleistocalyx operculatus*, and *Trewia nudiflora* for low elevation; T*erminalia alata*, *Diploknema butyracea* , and *Schima wallichii* for mid‐elevation; and *Quercus lanata*, *Quercus semecarpifolia*, and *Rhododendron arboreum* for high elevation.

### 3.3 Interspecific Traits Variation

The analysis of functional traits revealed clear interspecific variation across the studied tree species. Leaf thickness was higher in *Quercus semecarpifolia* (0.38 ± 0.03 mm) and lowest in *Trewia nudiflora* (0.13 ± 0.01 mm) (Table 4). LDMC reached its highest in *Diploknema butyracea* (1.46 ± 0.27 g/g) and its lowest in *T. nudiflora* (0.17 ± 0.04 g/g). TCH was maximum in *T. nudiflora* (3.81 ± 0.55 mg/g), whereas the minimum was recorded in *Rhododendron arboreum* (1.08 ± 0.89 mg/g). TN content was highest in *T. nudiflora* (0.65% ± 0.44%) and lowest in *D. butyracea* (0.23% ± 0.02%). Similarly, TP was highest in *S. robusta* (0.13% ± 0.02%) and lowest in *Quercus semecarpifolia* (0.07% ± 0.01%). The largest LA was found in *D. butyracea* (101.58 ± 32.59 cm^2^), whereas *R. arboreum* exhibited the smallest (27.91 ± 7.79 cm^2^). SLA was highest in *T. nudiflora* (106.59 ± 1.20 cm^2^/g) and was lowest in *Q. semecarpifolia* (50.40 ± 8.61 cm^2^/g) (Table 4).

**TABLE 4:** Mean ± standard deviation of leaf traits of the selected tree species.

Species	Thickness (mm)	LDMC (g/g)	TCH (mg/g)	TN (%)	TP (%)	LA (cm^2^)	SLA (cm^2^/g)
*Shorea robusta*	0.20 ± 0.01	0.28 ± 0.12	2.92 ± 1.79	0.59 ± 0.40	0.13 ± 0.02	83.18 ± 11.89	99.51 ± 16.21
*Cleistocalyx operculatus*	0.21 ± 0.03	0.36 ± 0.12	3.30 ± 0.93	0.45 ± 0.21	0.11 ± 0.02	66.99 ± 18.57	67.46 ± 5.12
*Trewia nudiflora*	0.13 ± 0.01	0.17 ± 0.04	3.81 ± 0.55	0.65 ± 0.44	0.11 ± 0.01	71.50 ± 5.30	106.59 ± 1.20
*Terminalia alata*	0.16 ± 0.03	0.59 ± 0.22	2.16 ± 0.59	0.29 ± 0.09	0.12 ± 0.01	43.47 ± 17.36	64.92 ± 5.85
*Diploknema butyracea*	0.19 ± 0.02	1.46 ± 0.27	3.36 ± 0.87	0.23 ± 0.02	0.11 ± 0.02	101.58 ± 32.59	82.07 ± 19.95
*Schima wallichii*	0.15 ± 0.01	0.65 ± 0.19	1.92 ± 0.99	0.38 ± 0.22	0.10 ± 0.02	37.34 ± 11.48	56.55 ± 15.80
*Quercus lanata*	0.22 ± 0.02	0.50 ± 0.13	2.06 ± 0.59	0.26 ± 0.10	0.08 ± 0.01	30.82 ± 3.35	58.18 ± 11.91
*Quercus semecarpifolia*	0.38 ± 0.03	0.46 ± 0.23	1.92 ± 0.99	0.23 ± 0.10	0.07 ± 0.01	28.38 ± 6.99	50.40 ± 8.61
*Rhododendron arboreum*	0.29 ± 0.02	0.52 ± 0.18	1.08 ± 0.89	0.29 ± 0.13	0.08 ± 0.01	27.91 ± 7.79	61.21 ± 8.70

### 3.4 Variation in Leaf Functional Traits Across Elevation Gradients

**FIGURE 2:**
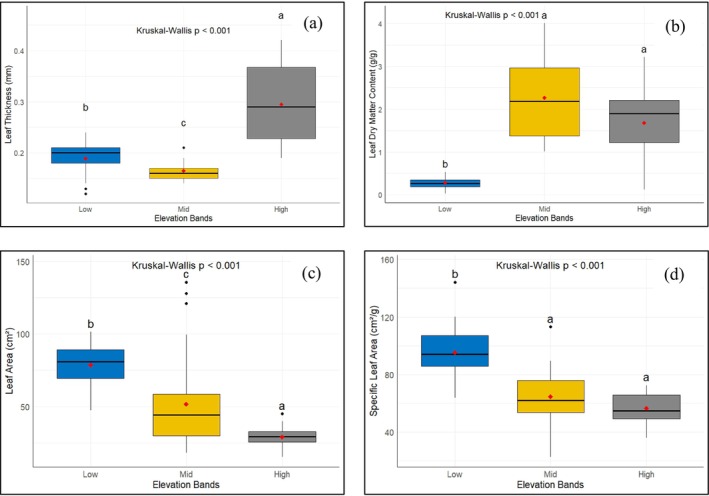
Leaf thickness (a), leaf dry matter content (b), leaf area (c), and specific leaf area (d) across elevation bands. Black horizontal lines indicate medians; red points represent mean values, and letters above boxes denote significant differences.

The Kruskal–Wallis tests revealed significant differences in all structural leaf traits across the three elevation groups. Leaf thickness significantly varied with elevation (*χ* ^2^ = 55.726, df = 2, *p* < 0.001), with Dunn's post hoc test showing significant differences among all pairs (Figure 2a). Leaf dry matter content also differed significantly (*χ* ^2^ = 50.786, df = 2, *p* < 0.001), but in pairwise comparison, the high–mid was not significantly different (*p* = 0.259) (Figure 2b). Leaf area exhibited a highly significant difference with elevation (*χ* ^2^ = 52.24, df = 2, *p* < 0.001), with all pairwise comparisons being significant (Figure 2c). Specific leaf area followed the same pattern, differing significantly across elevations (*χ* ^2^ = 46.87, df = 2, *p* < 0.001), except for the high–mid comparison, which was not statistically significant (*p* = 0.312) (Figure 2d).

**FIGURE 3:**
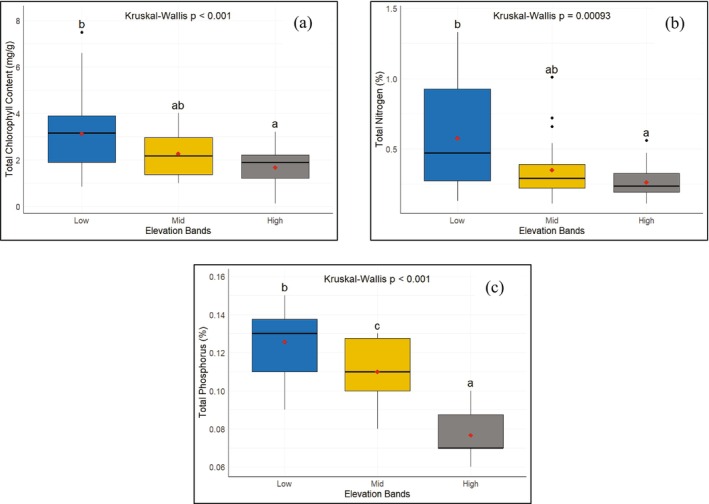
Total chlorophyll content (a), total nitrogen (b), and total phosphorus (c) across elevation bands. Black horizontal lines indicate medians; red points represent mean values, and letters above boxes denote significant differences.

Total chlorophyll content showed significant variation across elevation groups (*χ* ^2^ = 15.23, df = 2, *p* = 0.00049), with pairwise compared indicating that only the low–high pair differed significantly (*p* = 0.00029) (Figure 3a). Total nitrogen content followed a similar pattern (*χ* ^2^ = 13.96, df = 2, *p* = 0.00093) and only the low–high pair showed a significant difference (*p* = 0.00058), while the remaining pairs were not significant (Figure 3b). In contrast, total phosphorus displayed a much stronger elevation effect (*χ* ^2^ = 56.67, df = 2, *p* < 0.001), with all elevation pairs showing significant differences in pairwise comparisons (Figure 3c).

### 3.5 Multivariate Functional Trait Differentiation Along the Altitudinal Gradient

**FIGURE 4:**
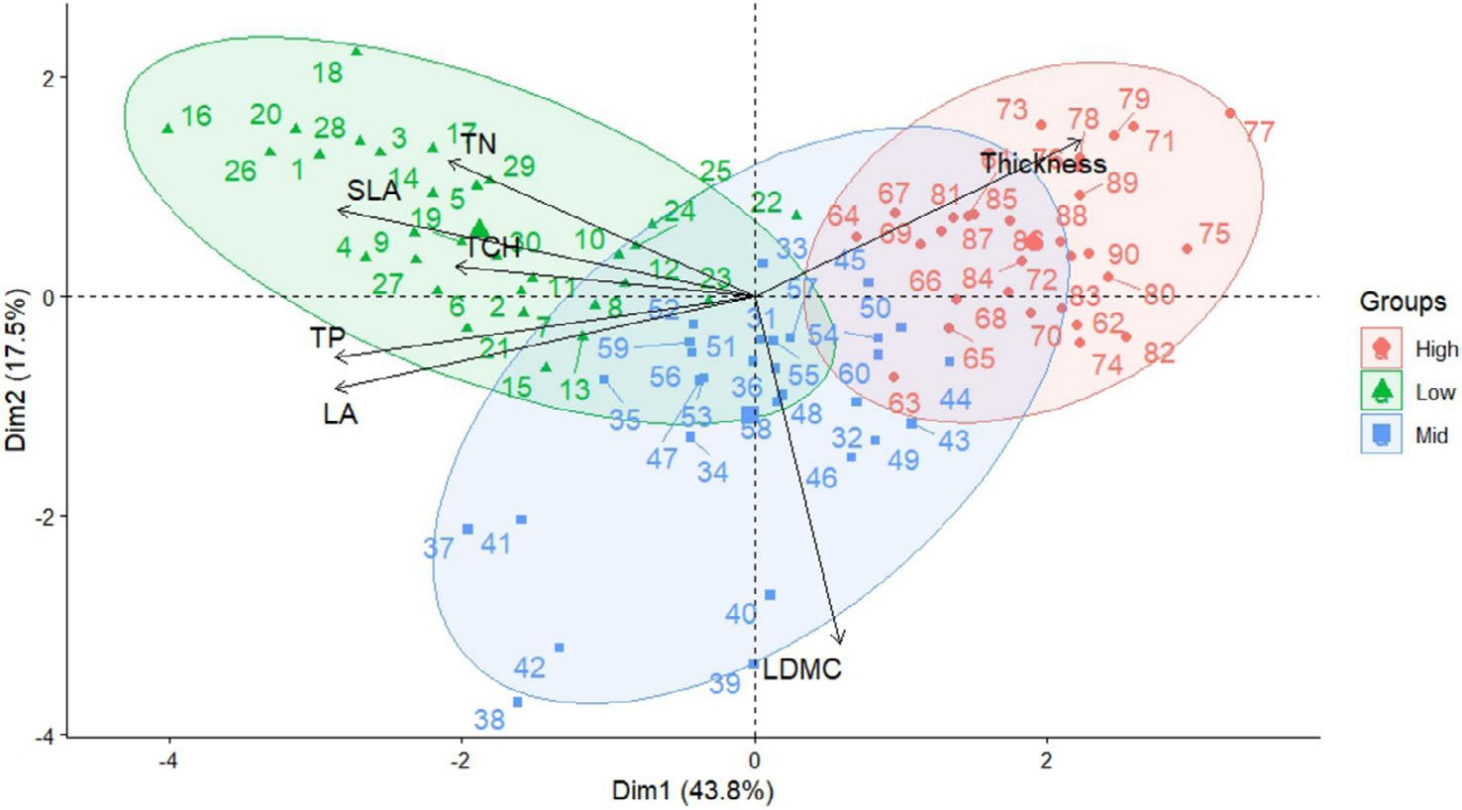
PCA biplot showing functional trait variation among elevation bands. Individual points represent plot‐level observations, colored according to elevation groups (low, mid, and high). Ellipses represent 95% confidence intervals of group distributions. Trait vectors indicate the contribution and direction of leaf functional traits to the principal components.

Principal component analysis (PCA) revealed clear differentiation in trait composition among elevation bands. The first two principal components explained 61.3% of the total variation, with PC1 accounting for 43.8% and PC2 accounting for 17.5% of the variation (Figure 4). The PCA indicated a strong separation between low‐ and high‐elevation plots along PC1. Low‐elevation plots were primarily associated with higher values of LA, SLA, TN, TP, and TCH, representing a more acquisitive trait strategy. In contrast, high‐elevation plots were positioned toward the positive side of PC1 and were associated with greater leaf thickness, indicating a shift toward more conservative resource‐use strategies.

Mid‐elevation plots occupied an intermediate position along PC1 but showed greater variation along PC2, partly associated with variation in LDMC. This suggests that mid‐elevation communities contain a wider range of functional strategies compared with low‐ and high‐elevation communities. PCA revealed a clear shift in functional trait composition across the altitudinal gradient, reflecting changes in plant resource acquisition and conservation strategies with elevation.

PERMANOVA revealed significant differences in overall functional trait composition among the three elevation bands (F_2_,_87_ = 35.77, *p* = 0.001), with elevation explaining 45.1% of the total variation in multivariate trait composition (*R* ^2^ = 0.451). Pairwise PERMANOVA comparisons showed significant differences among all elevation pairs, including low–mid (F = 21.39, *R* ^2^ = 0.269, *p* = 0.001), low–high (F = 61.01, *R* ^2^ = 0.513, *p* = 0.001), and mid–high (F = 29.53, *R* ^2^ = 0.337, *p* = 0.001). The strongest differentiation occurred between low‐ and high‐elevation communities, indicating substantial shifts in functional trait composition along the altitudinal gradient.

Multivariate dispersion analysis showed significant differences in within‐group trait variability among elevation bands (F_2_,_87_ = 5.68, *p* = 0.005). High‐elevation communities exhibited greater functional trait dispersion compared with low (*p* = 0.005) and mid elevations (*p* = 0.041), whereas low‐ and mid‐elevation communities did not differ significantly (*p* = 0.730). This suggests that high‐elevation communities contained a broader range of functional strategies among coexisting species.

### 3.6 Multivariate Patterns and Relationships Among Functional Traits

**FIGURE 5:**
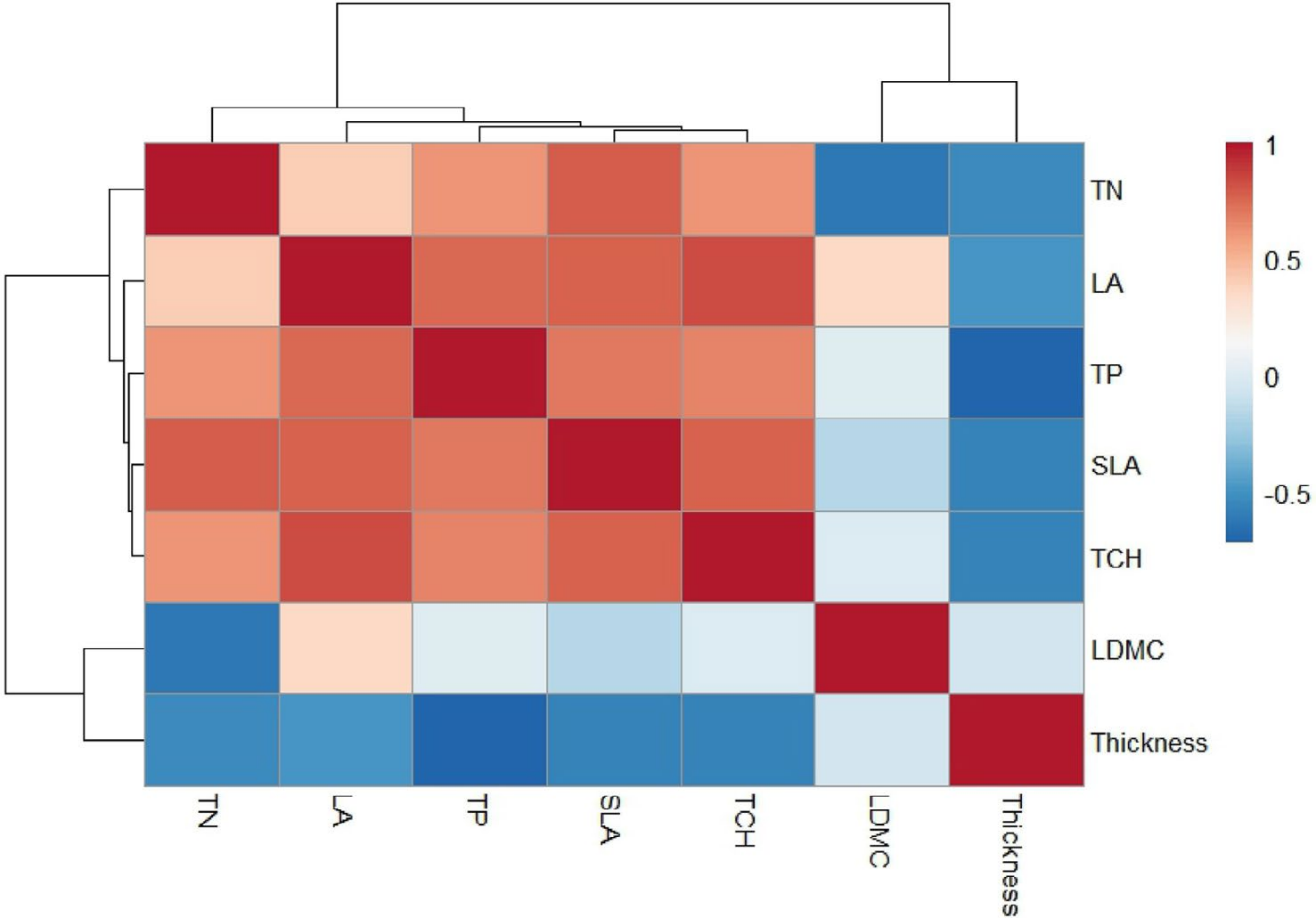
Correlation heatmap showing relationships among leaf traits. Red indicates positive and blue indicates negative correlations, and traits are clustered based on similarity.

The correlation heatmap revealed distinct patterns among the measured leaf traits. A strong, positive correlation was observed among the five traits, specifically SLA, TN, TP, LA, and TCH. These variables clustered together, indicating similar patterns of variation across samples. In contrast, LDMC and leaf thickness exhibited a strong positive correlation with each other but a negative correlation with SLA, TP, and TN. The hierarchical clustering grouped the traits into two main clusters: one comprising SLA, TP, LA, TN, and TCH, and the other comprising LDMC and Thickness (Figure 5).

### 3.7 Functional Trait–Biomass Relationships Along the Altitudinal Gradient

The average aboveground tree biomass (AGTB) varied considerably among species distributed across different elevation zones. The highest AGTB was recorded in the low‐elevation species *Shorea robusta* (20.46 kg m^−2^), followed by *Cleistocalyx operculatus* (13.51 kg m^−2^) and *Trewia nudiflora* (11.12 kg m^−2^). In contrast, species occurring at higher elevations, including *Rhododendron arboreum* (2.07 kg m^−2^), *Quercus lanata* (3.44 kg m^−2^), and *Schima wallichii* (4.68 kg m^−2^), showed comparatively lower biomass values. These patterns suggest that variation in biomass among species may reflect the combined effects of functional trait differences and environmental filtering associated with elevation.

**FIGURE 6:**
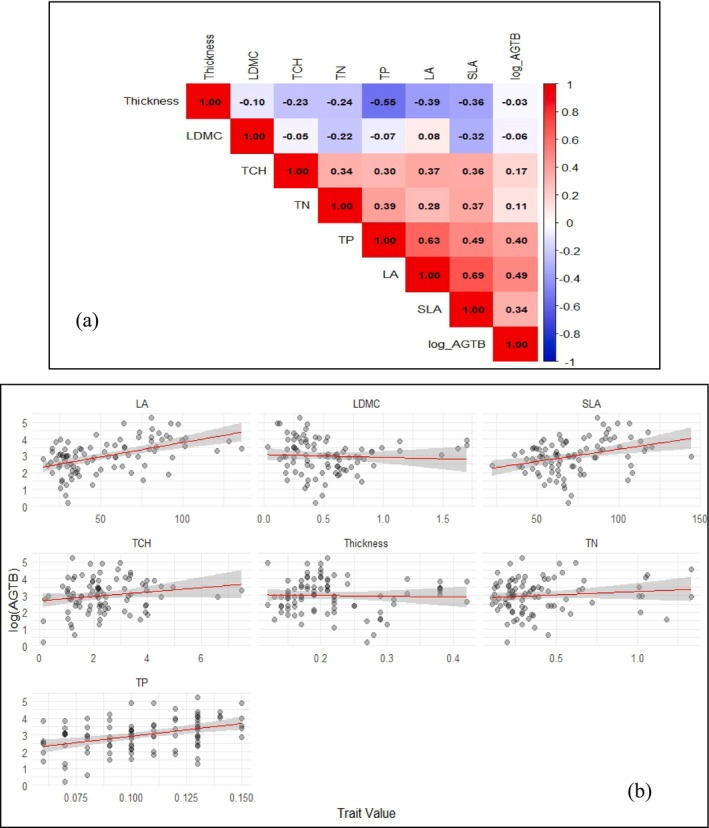
(a) Correlation matrix showing relationships between leaf traits and aboveground tree biomass across elevation bands. (b) Regression plots illustrating the relationships between log‐transformed aboveground biomass (log AGTB) and key leaf traits along the elevation gradient.

Correlation analysis showed that AGTB was moderately and positively associated with leaf area (*r* = 0.492) and total phosphorus (*r* = 0.398) (Figure 6a). SLA also showed a weak‐to‐moderate positive relationship with biomass (*r* = 0.340), indicating that species with greater light interception capacity and resource acquisition strategies tended to exhibit higher biomass. Total chlorophyll (*r* = 0.171) and total nitrogen (*r* = 0.114) showed weak positive associations, whereas leaf thickness (*r* = −0.034) and LDMC (*r* = −0.059) showed negligible relationships with AGTB (Figure 6a).

Linear regression analyses identified LA, SLA, and TP as significant predictors of AGTB (Figure 6b). LA showed a positive relationship with biomass (slope = 0.017, *p* < 0.001), explaining 23% of the variation in AGTB. TP also showed a significant positive relationship (slope = 15.27, *p* < 0.001; adjusted *R* ^2^ = 0.149), while SLA exhibited a weaker but significant positive effect (slope = 0.015, *p* = 0.001; adjusted *R* ^2^ = 0.105). In contrast, LDMC, leaf thickness, TCH, and TN were not significantly associated with biomass (*p* > 0.1). The biomass variation among species was associated with acquisitive traits, particularly larger leaf area and higher phosphorus concentration, while these relationships should be interpreted within the context of environmental variation along the altitudinal gradient.

### 3.8 Structural Equation Modeling

The Structural Equation Model showed a good fit to the data, with a nonsignificant chi‐square test (*χ* ^2^ = 6.473, df = 6, *p* = 0.372), indicating no significant difference between the observed and model‐implied covariance structures. Model fit indices further confirmed excellent fit, with high Comparative Fit Index (CFI = 0.997) and Tucker–Lewis Index (TLI = 0.993), and low Root Mean Square Error of Approximation (RMSEA = 0.030) and Standardized Root Mean Square Residual (SRMR = 0.038) (Table 5).

**TABLE 5:** Goodness‐of‐fit indices for the final structural equation model used in the trait–biomass relationship analysis.

Fit index	Value	Criteria for good fit	Fit status
Chi‐square (*χ* ^2^)	6.473	*p* > 0.05 (nonsignificant)	Good fit (*p* = 0.372)
Degrees of freedom (df)	6	—	—
Comparative fit index (CFI)	0.997	≥ 0.95	Excellent fit
Tucker‐Lewis index (TLI)	0.993	≥ 0.95	Excellent fit
RMSEA	0.03	≤ 0.05	Excellent fit
RMSEA 90% confidence interval	0.000–0.143	Upper limit ≤ 0.08 preferred	Marginal (upper CI > 0.08)
SRMR	0.038	≤ 0.08	Excellent fit

**FIGURE 7:**
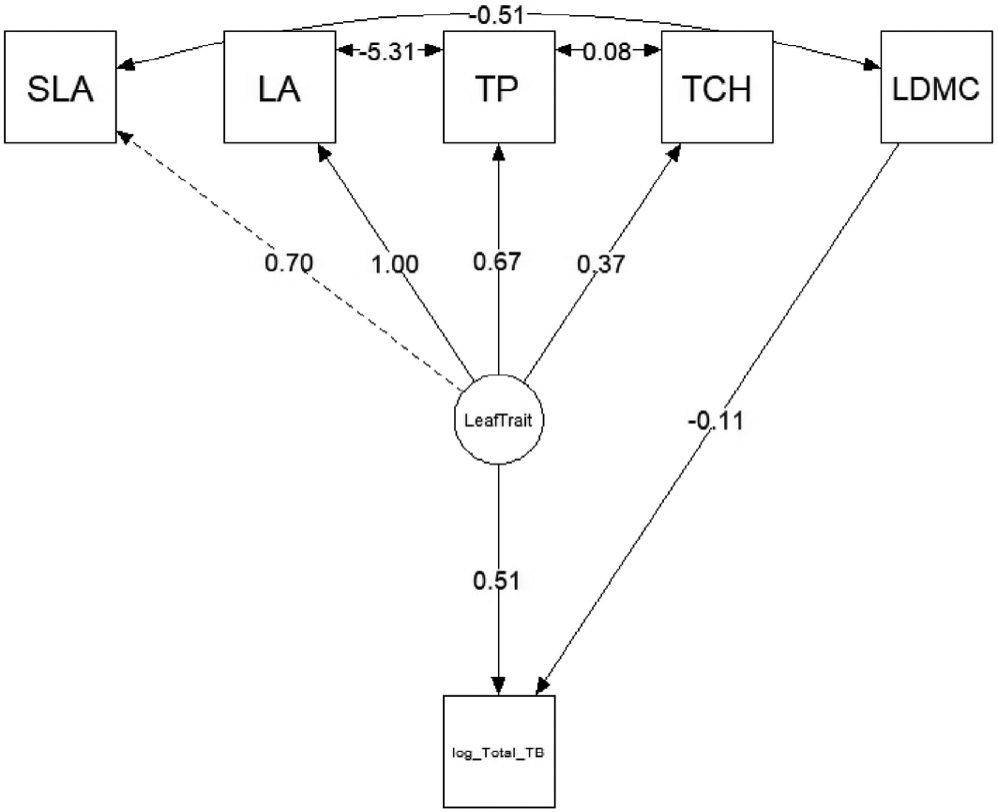
Structural equation model showing relationships between leaf traits and biomass accumulation. Arrows indicate path directions with standardized coefficients.

The latent construct LeafTrait was significantly represented by SLA, LA, TP, and TCH, indicating that these variables jointly reflect a common functional trait dimension. Among these, LA showed the strongest loading (0.997), followed by SLA (0.706) and TP (0.670), while TCH contributed moderately (0.370), suggesting variation in the strength of trait contributions to the latent functional axis (Figure 7).

The structural model indicated that LeafTrait had a significant positive effect on aboveground biomass (standardized coefficient = 0.504, *p* < 0.001), suggesting that increases in acquisitive leaf functional traits are associated with higher biomass accumulation. In contrast, LDMC showed a weak and nonsignificant negative effect on biomass (standardized coefficient = −0.097, *p* = 0.289), indicating that conservative traits had limited direct influence on biomass in this system.

The model further revealed a significant negative relationship between SLA and LDMC (standardized covariance = −0.512, *p* < 0.001), reflecting a clear trade‐off between resource acquisition and structural investment strategies. Other trait covariances (LA–TP and TP–TCH) were not significant after accounting for the latent trait structure, suggesting that their relationships are largely explained through the shared functional trait axis.

### 3.9 Relation Between Leaf Traits, Biomass, and Soil Parameters

The correlation analysis between soil parameters and leaf traits revealed several notable patterns. Leaf thickness was positively correlated with soil potassium (*r* = 0.60), organic matter (*r* = 0.44), and nitrogen (*r* = 0.43) but negatively with soil pH (*r* = −0.52). TN, TP, LA, and SLA were strongly positively associated with soil phosphorus (*r* = 0.67 for TN, 0.66 for TP, 0.66 for LA, 0.54 for SLA), clay content (*r* = 0.47 for TP, 0.50 for LA, 0.64 for SLA), and silt content (*r* = 0.40 for TP, 0.48 for LA, 0.66 for SLA), indicating that finer‐textured, phosphorus‐rich soils favor leaves with higher nutrient content and larger area. In contrast, sandy soils showed negative correlations with leaf thickness (*r* = −0.22), TP (*r* = −0.44), LA (*r* = −0.50), and SLA (*r* = −0.67). Electrical conductivity (EC) had mixed effects, with a moderate negative correlation with LDMC (*r* = −0.38) and positive correlations with TN (*r* = 0.34) and SLA (*r* = 0.33).

The correlation analysis between aboveground tree biomass and soil properties indicated generally weak relationships. Biomass showed a weak positive correlation with soil organic matter (*r* = 0.26) and very weak positive correlations with soil pH (*r* = 0.05), potassium (*r* = 0.13), and sand content (*r* = 0.04). Weak negative correlations were observed with nitrogen (*r* = −0.17), phosphorus (*r* = −0.22), clay content (*r* = −0.22), and electrical conductivity (*r* = −0.25), indicating that these soil parameters had minimal influence on biomass variation across the study sites.

### 3.10 Redundancy Analysis

**FIGURE 8:**
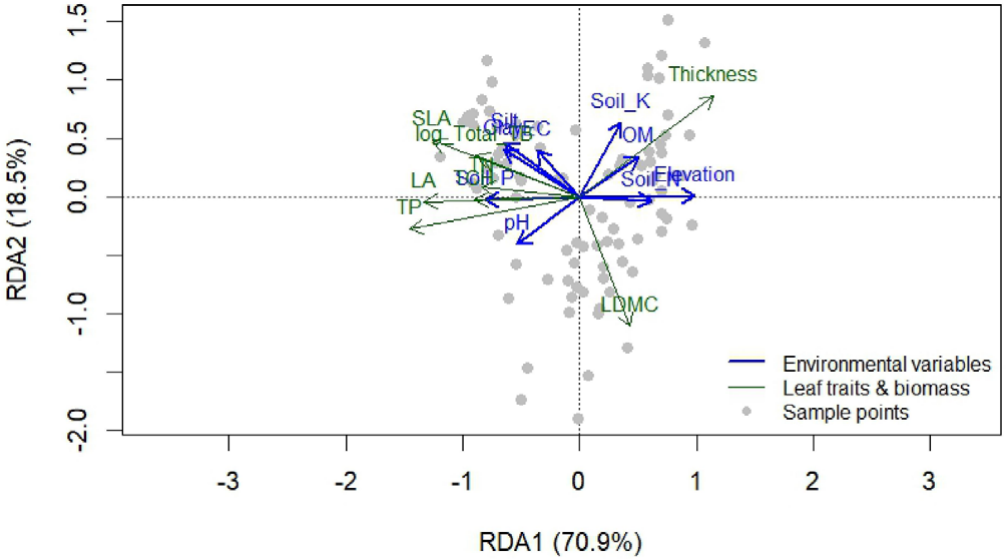
Redundancy analysis biplots illustrating the relationships between environmental variables and, leaf traits and aboveground biomass.

RDA showed that the first two axes explained 89.4% of the total constrained variation, with RDA1 accounting for 70.9% and RDA2 accounting for 18.5% (Figure 8). Elevation, soil organic matter (OM), soil potassium (Soil K), and EC were positively associated with leaf thickness and LDMC, indicating that higher elevation and nutrient‐rich soils favor conservative leaf traits. In contrast, SLA, LA, Total TB, TCH, and TN were negatively associated with elevation and were more closely related to soil phosphorus (Soil P), silt, and pH, suggesting that lower elevation conditions promoted acquisitive traits and higher biomass accumulation. Among environmental variables, elevation, OM, and Soil K showed comparatively stronger influence on trait variation, as indicated by their longer vectors in the ordination space (Figure 8). RDA and regression analyses showed that elevation and soil properties explained 45.4% of the variation in leaf functional traits (adjusted *R²* = 0.454) and 30.8% of the variation in above‐ground biomass (adjusted *R²* = 0.308), respectively.

## 4 Discussion

Species richness and diversity often exhibit distinct patterns along elevation gradients. Globally, a hump‐shaped or unimodal relationship, where diversity peaks at mid‐elevations, is one of the most commonly reported patterns (Rahbek 2005). In the present study, species richness (q0) and Hill number q1 also peaked at mid‐elevation, indicating greater species diversity and coexistence in intermediate zones. The higher diversity observed at mid‐elevations may be associated with favorable environmental conditions, including moderate temperature, moisture availability, and habitat heterogeneity, which promote species establishment and coexistence (Colwell and Lees 2000; Rahbek 2005). In addition, mid‐elevation zones often represent transitional environments where species from different phytogeographic affinities overlap, increasing habitat heterogeneity and allowing coexistence of species from both lower and higher elevations. Variation in temperature and precipitation along elevation gradients further influences species distribution and community assembly, contributing to the commonly observed mid‐elevation diversity peak (Bhattarai and Vetaas 2006; McCain and Grytnes 2010). According to Tuomisto (2010), higher diversity values reflect greater biodiversity and ecosystem complexity, whereas lower values indicate reduced species diversity and dominance by a few taxa.

Similarly, Hill number q1, representing the effective number of common species, was highest at mid elevation, indicating greater species diversity and community heterogeneity in this zone. In contrast, low elevation exhibited lower q1 and q2 values, suggesting dominance by a few abundant species, particularly *Shorea robusta* , which is characteristic of tropical Sal forests in Nepal (Malla and Acharya 2018). This observation aligns with Lou Jost et al. (2010), who noted that diversity declines with increasing species dominance. Species evenness increased with elevation, indicating a more balanced distribution of species abundances at higher altitudes. Such patterns may be associated with harsher environmental conditions, including lower temperatures, shorter growing seasons, and nutrient‐poor soils, which reduce competitive exclusion and limit species dominance (Bhattarai and Vetaas 2003; Körner 2007).

Variation in species diversity along the altitudinal gradient was closely associated with changes in soil properties and functional trait expression. Mid‐elevation sites exhibited relatively higher diversity and functional variability, possibly due to more favorable environmental conditions and greater habitat heterogeneity. Soil properties such as moisture retention, organic matter accumulation, and nutrient availability may have contributed to shifts in plant ecological strategies, reflected in variation in traits such as SLA and LDMC. These trait adjustments likely influenced biomass accumulation and vegetation structure across the gradient (Körner 2007; Sundqvist et al. 2013; Díaz et al. 2016). However, functional trait responses should not be considered the direct consequence of individual environmental variables alone. Instead, traits reflect the combined influence of multiple interacting factors, including climate, soil conditions, topography, and species‐specific ecological strategies. Therefore, relatively weak relationships between individual soil variables and traits may still represent ecologically meaningful responses under complex natural conditions (Violle et al. 2007; Laughlin 2014).

Even within the same altitudinal band, different species exhibit distinct leaf trait variations because trait expression is governed by species‐specific genetic strategies, physiological adaptations, and ecological trade‐offs. Species differ in resource‐use efficiency and photosynthetic capacity, leading to significant interspecific variation in traits despite similar environmental conditions (Reich et al. 2003; Wright et al. 2004; Freschet et al. 2010). Fajardo and Siefert (2018) further highlight that trait variation arises from both genetic differences and intraspecific plasticity, emphasizing the combined influence of evolutionary history and environmental filtering. Therefore, variation among co‐occurring species within the same band reflects their inherent functional strategies shaped by both evolutionary history and microhabitat conditions (Díaz et al. 2016).

Leaf functional traits of dominant species showed clear elevation‐driven patterns, reflecting species‐specific strategies along environmental gradients. Lowland species such as *Shorea robusta* , *Cleistocalyx operculatus*, *Trewia nudiflora*, *Terminalia alata*, and *Schima wallichii* exhibited higher LA, SLA, chlorophyll content, and TN and TP concentrations, indicating resource‐acquisitive strategies favored under warm temperatures, fertile soils, and high light availability (Wright et al. 2004; Poorter et al. 2009). Chlorophyll content was highest in lowland species, supporting higher photosynthetic capacity, whereas declines at mid‐ and high elevations reflect the combined effects of colder temperatures, nutrient limitation, and exposure to strong UV radiation, which restricts photosynthesis in alpine species (Richardson et al. 2002; Zhu et al. 2020; Yang et al. 2025). These patterns align with reports that SLA, LA, and leaf nutrient concentrations decrease with elevation due to physiological and environmental constraints (Liu et al. 2010; Peppe et al. 2011; Lusk et al. 2018; Dhiman et al. 2023; Ji‐Shi et al. 2024).

In contrast, high‐elevation species such as *Rhododendron arboreum* and *Quercus* spp. exhibited thicker leaves, higher LDMC, and lower SLA, LA, TN, and TP, demonstrating resource‐conservative strategies suited to cold, nutrient‐poor, and high‐UV environments (Niinemets 2001; Körner 2007; Pérez‐Harguindeguy et al. 2013; Read et al. 2014). Increased leaf thickness with elevation is a widely reported adaptation to resist cold, aridity, and UV damage (Henn et al. 2018; Zhang et al. 2024). Mid‐elevation species such as *Diploknema butyracea* displayed intermediate biomass and notably high LDMC, reflecting transitional strategies where local microclimate and disturbance shape trait expression as strongly as elevation itself (Laughlin 2011; Ye et al. 2024).

These trait variations collectively align with LES, which characterizes global trade‐offs between rapid resource acquisition and resource conservation (Wright et al. 2004; Reich 2014; Díaz et al. 2016). Strong positive correlations among SLA, LA, TN, TP, and chlorophyll reflect acquisitive strategies maximizing photosynthetic gain and growth (Poorter et al. 2009), whereas positive associations between LDMC and leaf thickness reflect conservative strategies emphasizing structural investment, longevity, and stress tolerance (Onoda et al. 2017).

Biomass patterns reflected these functional strategies. Lowland species with high SLA, high LA, and nutrient‐rich leaves showed greater biomass accumulation, which may reflect the combined influence of acquisitive functional strategies and favorable environmental conditions at lower elevations (Moser et al. 2007; Bruelheide et al. 2018). High‐elevation species, limited by short growing seasons, poor soils, and strong climatic stress, accumulated lower biomass, consistent with conservative strategies prioritizing survival over productivity (Körner 2007; Poorter et al. 2009). Mid‐elevation species such as *Diploknema butyracea* showed intermediate biomass, reflecting mixed strategies along the gradient. Correlation patterns further confirmed that resource‐acquisitive traits (SLA, LA, TN, TP) positively influenced biomass, whereas conservative traits (leaf thickness, LDMC) showed weak or negative relationships, reinforcing the functional trade‐off central to the LES (Wright et al. 2004; Reich 2014; Díaz et al. 2016).

Soil properties contributed to variation in functional traits and biomass accumulation; however, their effects should be interpreted within the broader context of interacting climatic conditions, topographic variation, and species‐specific ecological strategies along the altitudinal gradient. Nutrient‐rich, fine‐textured soils (high in phosphorus, clay, and silt) favored resource‐acquisitive traits, including larger, nutrient‐rich leaves and higher SLA, enhancing photosynthetic efficiency and growth (Reich et al. 2007; Fortunel et al. 2009). Coarse, nutrient‐poor soils promoted conservative traits, with smaller, thicker, and denser leaves adapted to resource limitation (Laughlin 2011). EC reflects dissolved ions and general soil chemical conditions rather than direct nutrient availability or salinity stress in forest soils (Brady and Weil 2016). Therefore, its relationship with functional traits should be interpreted as an indication of variation in soil chemical environments rather than a direct salinity response (Parida and Das 2005; Munns and Tester 2008). The slight positive relationship with soil organic matter aligns with previous studies showing that organic matter improves nutrient availability, enhances soil structure, and increases water retention, thereby supporting tree growth and productivity (Lal 2005; Augusto et al. 2017). However, the weak effect (*r* = 0.26) indicates that while soil fertility is beneficial, biomass accumulation is strongly influenced by species‐specific traits such as wood density and leaf economic strategies, which determine how efficiently trees convert resources into standing biomass (Fyllas et al. 2009; Poorter et al. 2016). The relationship between nitrogen and functional traits should be interpreted cautiously because this study measured total nitrogen rather than plant‐available nitrogen. Although total nitrogen reflects general soil fertility, plant responses are more directly influenced by available nitrogen forms in the soil. Previous studies have shown that nitrogen availability strongly influences leaf economic traits such as SLA and LDMC, thereby affecting photosynthetic capacity and biomass accumulation along elevational gradients (Reich et al. 1997; Ordoñez et al. 2009).

The SEM showed an excellent fit to the data with fit indices meeting established criteria for good model fit (CFI = 0.997; TLI = 0.993; RMSEA = 0.030; SRMR = 0.038), indicating that the hypothesized relationships between leaf traits and biomass adequately represent the observed data (Hu and Bentler 1999; Schreiber et al. 2006). The latent variable LeafTrait, represented by LA, SLA, TP, and TCH, had the highest loading for LA (0.997), suggesting its dominant role in the trait–biomass relationship. LeafTrait positively predicted above‐ground biomass (standardized coefficient = 0.504, *p* < 0.001), while LDMC had a nonsignificant negative effect (−0.097, *p* = 0.289), highlighting that resource‐acquisitive traits drive productivity, whereas conservative traits contribute less (Wright et al. 2004; Poorter et al. 2009; Reich 2014; Díaz et al. 2016). The negative covariance between SLA and LDMC (−0.512, *p* < 0.001) reflects the trade‐off between acquisitive and conservative strategies. These findings are consistent with previous trait‐based studies showing that leaf traits mediate ecosystem functioning along environmental gradients (Fortunel et al. 2009), supporting the Mass Ratio Hypothesis (Grime 1998) and the LES framework (Wright et al. 2004). The SEM results indicate that aboveground biomass is primarily driven by an integrated leaf trait strategy rather than isolated trait effects, highlighting the coordinated role of plant functional traits in regulating ecosystem productivity along the altitudinal gradient.

Therefore, while the observed trait–biomass relationships are broadly consistent with established ecological frameworks such as the leaf economics spectrum, our findings indicate that these patterns are also influenced by altitudinal variation in species composition and soil properties. This suggests that both environmental filtering and species turnover contribute to the observed functional patterns, highlighting the importance of local edaphic and climatic contexts in shaping trait expression and biomass accumulation. These elevation‐dependent differences in functional strategies have important implications for forest conservation and restoration, as management approaches should consider the distinct ecological characteristics of low‐, mid‐, and high‐elevation forests. In particular, conserving mid‐elevation forests may be important for maintaining high species diversity and functional heterogeneity, while high‐elevation forests require protection due to the presence of specialized species adapted to environmentally stressful conditions (Körner 2007; Díaz et al. 2016). However, a key limitation of this study is that within‐species trait variation was not explicitly separated from species turnover effects due to data constraints. Future studies incorporating community‐weighted mean (CWM) traits or intraspecific trait variation would provide a more robust understanding of these mechanisms and help better disentangle trait–environment relationships along environmental gradients.

## 5 Conclusion

The study demonstrated clear variation in species diversity, leaf functional traits, soil properties, and aboveground biomass along the altitudinal gradient in central Nepal. Mid‐elevation forests supported relatively higher species diversity, while functional trait composition and biomass patterns varied across environmental conditions along the gradient. Functional traits associated with resource acquisition, particularly specific leaf area and leaf nutrient content, were positively related to biomass. In contrast, traits linked to structural investment and stress tolerance exhibited comparatively weaker associations. By integrating functional trait analysis with soil and environmental variables across a broad elevational gradient, this study provides insight into how environmental filtering influences vegetation structure and ecosystem functioning in tropical–subtropical Himalayan forests. These relationships should be interpreted within the scope of the measured environmental variables and dominant species included in the analysis. Nevertheless, the findings highlight the value of trait‐based approaches for understanding vegetation responses to environmental gradients and may support forest conservation, ecological restoration, and sustainable management of ecologically sensitive Chure landscapes under changing environmental conditions.

## Author Contributions

**Shakti Raj Giri:** conceptualization (equal), data curation (equal), formal analysis (lead), investigation (lead), methodology (equal), software (equal), writing – original draft (lead), writing – review and editing (equal). **Purnima Regmi:** data curation (equal), investigation (equal), methodology (equal), software (equal), writing – review and editing (equal). **Rashila Deshar:** conceptualization (equal), funding acquisition (equal), project administration (equal), validation (equal), writing – review and editing (equal). **Ramesh Prasad Sapkota:** conceptualization (equal), formal analysis (equal), funding acquisition (equal), methodology (equal), project administration (lead), supervision (lead), validation (equal), writing – review and editing (lead).

## Funding

This work was supported by the University Grants Commission (UGC), Nepal (Grant No. FRG‐80/81‐S&T‐06).

## Disclosure

*Statement on Inclusion*: This study was conducted in Chitwan District, Nepal, and includes researchers based in the country where the research was carried out. All authors were involved from the early stages of study design through data collection, analysis, and interpretation, ensuring that local ecological context and perspectives were incorporated throughout the research process. Relevant literature published by scientists working in Nepal and the broader South Asian region were reviewed and cited where appropriate. The research was conducted with the necessary permissions and in consideration of local environmental conditions and management priorities. The findings are intended to contribute to ecological understanding and forest management efforts within the region.

## Conflicts of Interest

The authors declare no conflicts of interest.

## Supporting information


**Data S1:** ece374171‐sup‐0001‐Supinfo.xlsx.

## Data Availability

The data that support the findings of this study are available in the Supporting Information of this article.
